# Ultrasound for Early Detection of Melanoma Lymph Node Metastasis and Wire Localization of Nonpalpable Findings: A Case Report

**DOI:** 10.1155/crdm/7643002

**Published:** 2026-09-16

**Authors:** Susanne Bauer, Mark Berneburg, Bernadett Kurz, Melissa Radspieler, Konstantin Drexler, Sebastian Haferkamp, Julian Kögel

**Affiliations:** ^1^ Department of Dermatology and Allergology, University Hospital Regensburg, Regensburg, Germany, uniklinikum-regensburg.de

**Keywords:** lymph node metastases, melanoma, sonography, staging, wire localization

## Abstract

**Background:**

Malignant melanoma is the skin cancer with the highest mortality rate. Accurate detection of regional lymph node metastases is crucial for staging and treatment planning. Small‐volume nodal disease may remain occult on cross‐sectional imaging, while ultrasound can help detect particularly small regional lymph node metastases. The increasing role of neoadjuvant therapies further emphasizes the importance of early detection.

**Case:**

We present the case of a 63‐year‐old patient with a 16‐mm‐thick, ulcerated melanoma of the right thigh, initially classified as Stage IIC. Clinical examination and CT/MRI showed no evidence of metastatic disease. However, ultrasound identified a suspicious, nonpalpable right inguinal lymph node with malignant sonographic features. Due to its proximity to the femoral vessels, CT‐guided wire localization was performed prior to surgical excision. Histopathology confirmed an 8‐mm lymph node metastasis, resulting in upstaging to Stage IIIC (pN1b). Adjuvant PD‐1 inhibition was initiated for BRAF wild‐type melanoma.

**Conclusion:**

This case highlights the potential of ultrasound to detect small‐volume, radiologically occult lymph node metastases and thereby substantially alter melanoma staging and therapeutic management. In the era of neoadjuvant treatment strategies, early and accurate detection of regional metastatic disease may become increasingly important. Preoperative wire localization represents a feasible option for precise surgical removal of non‐palpable lymph node metastases.

## 1. Introduction

Malignant melanoma is one of the skin cancers with the highest metastases and mortality rate [1]. When a mole is clinically suspicious, complete excision of the primary lesion with a narrow surgical margin is recommended to establish the diagnosis while achieving complete tumor removal. Once the diagnosis of malignant melanoma is confirmed, further treatment depends on the tumor stage according to the TNM classification, with Breslow thickness representing the most important prognostic factor [1].

In early stages of melanoma (IB–IIB), ultrasound examination of the regional lymph nodes and serological determination of tumor progression parameters should be performed [1]. The likelihood of sentinel lymph node metastasis increases with greater Breslow thickness. Especially in Stage IIC, there is a similar risk of metastases, recurrence and melanoma specific survival as in patients with initial locoregional metastases (Stage III) [1, 2]. In contrast patients with cancer Stages IIB and below have a longer recurrence‐ and metastasis‐free survival rate [2]. Consequently, current guidelines recommend extended cross‐sectional imaging (CT, MRI, or PET‐CT) for patients with Stage IIC disease or higher [1].

In the following, we present the case of a patient with malignant melanoma, in which ultrasound examination identified a suspicious lymph node.

## 2. Case

A 63‐year‐old patient with a new diagnosis of malignant melanoma on the right thigh was admitted to our clinic (Figure 1). The skin lesion had been increasing in size for years. The initial tumor stage according to the AJCC 8th edition showed a malignant melanoma, Stage IIC, pT4b (tumor thickness of 16 mm, positive ulceration status), cNx, cMx, R2, ED 04/2025. Palpation of the lymph nodes and the rest of the full body examination revealed no abnormalities. In accordance with the German guidelines, cross‐sectional imaging with a full body CT and MRI of the head was performed and ruled out hematogenous or lymphogenous metastases (Figure 2A). However, high‐resolution ultrasound identified a suspicious right inguinal lymph node characterized by malignant sonographic features (Figure 2B,C). We performed surgery on the right thigh, achieving a total safety margin of 2 cm. During the same procedure, the sonographically suspicious lymph node was excised. Due to the not possible palpation of the lymph node and its proximity to the femoral vessels, preoperative CT‐guided wire marking was performed. Histopathological analysis confirmed an 8‐mm lymph node metastasis, immunohistochemically showing a moderate to strong expression of HMB45 while details on the mitosis rate are missing (Figure 3). Based on the detection of nodal involvement, the tumor stage was revised to Stage IIIC, pT4b (tumor thickness of 16 mm, positive ulceration status), pN1b, cM0, R0, ED 04/2025. As molecular testing demonstrated a BRAF wild‐type melanoma, adjuvant treatment with a PD‐1 immune checkpoint inhibitor pembrolizumab was initiated. The patient is currently being treated by an oncologist nearby. According to the latest medical report, the disease has shown progression with pulmonary metastases, so systemic therapy was escalated to ipilimumab/nivolumab.

**FIGURE 1:**
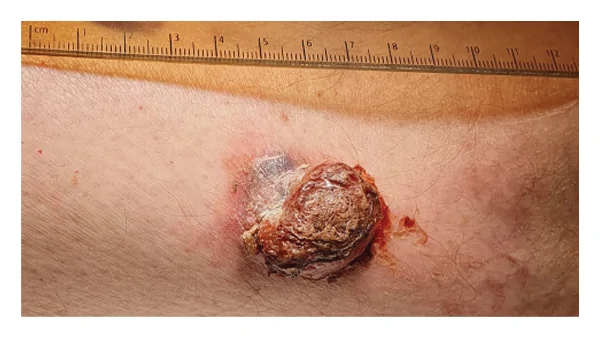
Initial preoperative situation: 3 cm large, centrally ulcerated, exophytic growing tumor at the right lateral thigh (patient photo documentation).

**FIGURE 2:**
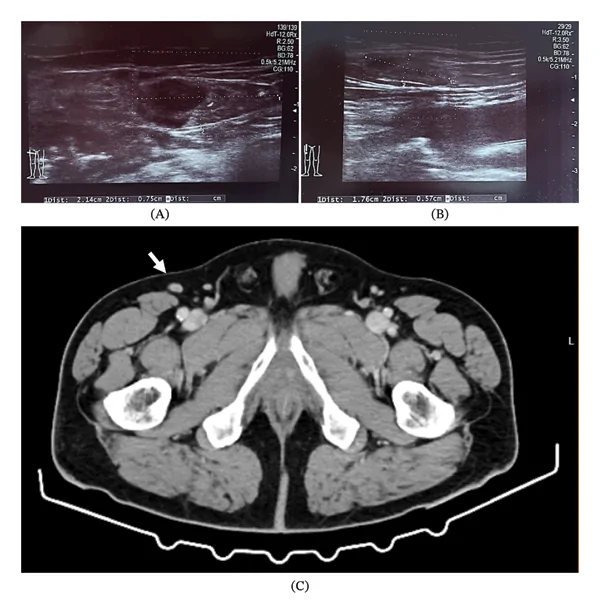
(A, B) Ultrasound findings: 2.14 × 0.75 × 1.76 cm diameter, longitudinal oval lymph node. Inhomogeneous internal structure with eccentric shifting of the echoic hilum. Echo‐poor area within the regular pattern of a lymph node. Irregular widening of the parenchymal band. The doppler sonography shows a still preserved central vascularization. (C) CT findings: slightly accentuated inguinal lymph node (arrow) on the right side.

**FIGURE 3:**
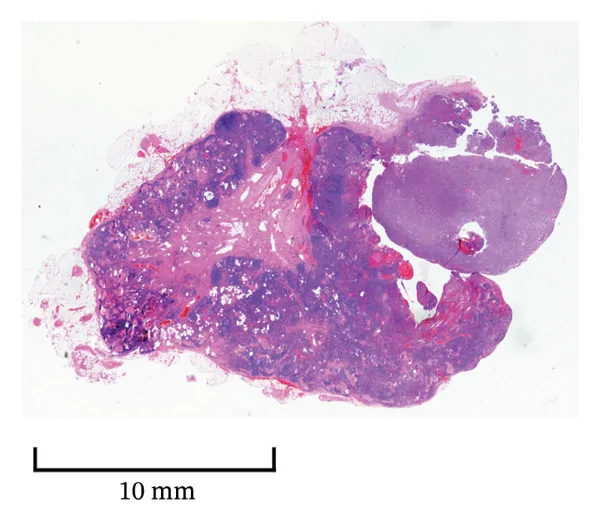
Histopathological findings: centrally fibrotic lymph node with a solid tumor metastasis measuring 8 mm with nuclear pleomorphism, enlarged, partly hyperchromatic, partly vesicular nuclei with cherry‐red macronuclei, increased mitotic activity, and blurred cell borders with an overall eosinophilic to bright eosinophilic cytoplasm. The tumor cells appear sporadically with a brownish pigment (hematoxylin–eosin, bars = 10 mm). In immunohistochemical analysis, the malignant neoplastic infiltrates show a moderate to strong expression of HMB45.

## 3. Discussion

The extent of initial staging diagnostics depends on the stage of the primary tumor. Therefore, the confirmation or exclusion of metastases is crucial for further treatment decisions [1]. In addition to sonography of the regional lymph nodes, CT, MRI, or PET CT are recommended for patients with advanced stage disease [1].

Sonography is a fast, inexpensive and noninvasive method for visualizing lymph nodes [1]. Diagnostic accuracy can be improved by combining multiple sonographic criteria. In particular, peripheral vascularization, loss of the central echogenic hilum, and a rounded (“balloon‐shaped”) lymph node morphology seem to be highly sensitive predictors of metastatic involvement [3]. In the present case, the most suspicious findings were a localized echo‐poor area within the regular pattern of a lymph node as well as asymmetric cortical widening. Both criteria have been described as sonographic signs of early lymph node metastasis [4].

The early detection of lymph node metastasis is becoming increasingly relevant in the management of melanoma [1]. Based on the results of the MSLT II and DeCOG SLT trials, completion lymph node dissection is no longer routinely recommended for patients with sentinel lymph node micrometastases and careful imaging follow‐up has gained prominence [5]. Among patients with resectable, macroscopic stage III melanoma, the NADINA study demonstrated longer event‐free survival if initially receiving neoadjuvant therapy consisting of ipilimumab plus nivolumab, followed by surgery and response‐dependent adjuvant therapy [6]. In our patient, the initial melanoma was classified as Stage IIC, pT4b (tumor thickness of 16 mm, positive ulceration status), cNx, cMx, R2, ED 04/2025, with no evidence of metastasis on cross‐sectional imaging. However, regional ultrasound identified a lymph node that was considered suspicious. The later histological confirmation results in an upstaging to Stage IIIC, pT4b (tumor thickness of 16 mm, positive ulceration status), pN1b, cM0, R0, ED 04/2025. This finding highlights the potential discrepancy between radiological staging and sonography in the detection of regional metastasis, particularly in the case of small‐volume lymph node metastasis. In retrospect, the histologically confirmed Stage IIIC disease places the patient within the broader clinical context addressed by the NADINA trial although the lesion in our patient was considerably smaller and not radiologically apparent and, therefore, does not directly correspond to the macroscopic Stage III disease population enrolled in the trial. Therefore, the study’s findings cannot be directly applied to this specific case. Nonetheless, early detection of lymph node metastasis is likely to become increasingly important as neoadjuvant treatment strategies continue to expend.

In the case of nonpalpable lymph nodes, surgical treatment is often difficult to perform. Wire marking of axillary lymph nodes has become an established technique in patients with breast cancer and confirmed lymph node metastasis. The technique has shown an improve intraoperative localization and complete resection rates [7]. In our patient, ultrasound‐guided wire placement was not possible due to the proximity of the suspicious lymph node to the femoral artery and vein and the required horizontal trajectory of the localization wire. Therefore, CT‐guided wire localization via a ventral approach was selected as a safe alternative, minimizing the risk of vascular injury while allowing precise preoperative localization. The marking was performed immediately before surgery by an interventional radiologist. For the procedure, the patient was placed in the supine position. The target lymph node was initially identified using the native planning scan. After sterile preparation of the procedural field, local anesthesia was administered with 1% Xylonest. The lymph node was subsequently localized and marked using a Bard DuaLok breast localization wire (Bard Peripheral Vascular, Inc., Tempe, AZ, USA) under image guidance. The wire localization was completed without complications. Although postoperative imaging was not considered necessary, temporary immobilization between wire placement and surgery may help reduce the risk of wire displacement.

## 4. Conclusion

Ultrasound diagnostics is a fast, noninvasive, and efficient method for the detection of regional lymph node metastases. In everyday clinical practice, specific sonographic criteria enable the identification of lymph node metastases with a high diagnostic sensitivity. When suspicious lymph nodes are not clinically palpable, preoperative wire localization can be considered to accurate intraoperative identification and surgical excision. Early detection of lymph node metastasis will become even more important in the era of risk‐adapted follow‐up strategies and the growing role of neoadjuvant systemic therapies.

## Funding

No funding was received for this manuscript.

## Disclosure

The authors declare no conflicts of interest.

## Ethics Statement

Consent for the publication of the patient photographs and medical information was provided by the authors at the time of article submission to the journal stating that the patient gave consent for his photographs and medical information to be published in print and online and with the understanding that this information may be publicly available. Formal approval by an ethics committee was not required due to the complete anonymization of the data and the patient’s consent to publication. The study conducted in accordance with the 1964 Declaration of Helsinki and its later amendments.

## Consent

The patients provided written informed consent for the publication of this report, including the images.

## Conflicts of Interest

The authors declare no conflicts of interest.

## Data Availability

Susanne Bauer has full access to all the data related used in this study, but said data cannot be made publicly available for reasons of patient privacy.
